# Clear Cell Myoepithelial Carcinoma Arising from the Hard Palate with Metastasis to the Lungs

**DOI:** 10.1155/2019/3863270

**Published:** 2019-01-06

**Authors:** Jiayun Fang, Amanda Kornfield, Alex Clavijo, Shikhar Vyas, Alison Ulbrandt, Carsten Schroeder, J. Kenneth Byrd, Daniel Kleven

**Affiliations:** ^1^School of Medicine at Medical College of Georgia at Augusta University, Augusta, GA, USA; ^2^Department of Pathology at Medical College of Georgia at Augusta University, Augusta, GA, USA; ^3^Department of Radiology at Medical College of Georgia at Augusta University, Augusta, GA, USA; ^4^Department of Thoracic Oncology Surgery at Medical College of Georgia at Augusta University, Augusta, GA, USA; ^5^Department of Head and Neck Surgery at Medical College of Georgia at Augusta University, Augusta, GA, USA

## Abstract

Myoepithelial carcinoma is an uncommon tumor of the salivary glands, most commonly the parotid gland. Clear cell myoepithelial carcinoma is a rare variant with an aggressive behavior. Here, we describe a case of clear cell myoepithelial carcinoma arising from the hard palate in an elderly male who underwent resection of the tumor and postop radiation. Posttreatment imaging demonstrated bilateral pulmonary nodules and a C2 body lesion concerning for metastasis. Biopsy of the lung lesions revealed a monomorphous population of optically clear cells with hyperchromatic and pleomorphic nuclei which were morphologically similar to the prior resection specimen. There are few reported cases of clear cell myoepithelial carcinoma arising from the hard palate, and there are even fewer reports on metastases to the lungs. Due to the low number of reported cases, prognosis and treatment of this neoplasm is not well defined.

## 1. Introduction

Myoepithelial carcinoma, also known as malignant myoepithelioma, is a rare neoplasm that accounts for less than 2% of salivary gland tumors [1]. It forms a painless, slow-growing mass that most commonly arises from the palate and major salivary glands like the parotid [2, 3], although it can arise from the minor glands of the oral mucosa [4, 5]. In a 2015 study involving 29 patients over 10 years, the most common sites were the parotid gland, submandibular gland, and palate [6]. Rarely, myoepithelial carcinomas can arise in locations such as the lung [7] and sinonasal tract [8, 9]. There are a diverse number of tumor cell types seen in this neoplasm, and most cases demonstrate multiple cell types such as epithelioid, clear cell, plasmacytoid, spindled, and mixed [6]. Clear cell tumor cells with myoepithelial features are a rare subtype of myoepithelial carcinoma. These neoplasms are infiltrative and cause local destruction to nearby tissues, most commonly the adjacent bone. Metastasis is uncommon but has been seen in late stages of disease [4]. In this report, we present a case of clear cell myoepithelial carcinoma (CMCC) that arose from the left maxillary gland in an elderly man, with subsequent metastasis to the lungs.

## 2. Case Report

A 65-year-old African American male presented to the clinic with a painless hard palate mass that had slowly enlarged over a period of 7 months. The mass was associated with bleeding, dysphagia, and dysphonia. The patient's medical and family history were unremarkable. On physical exam, a pink exophytic lesion was easily visualized on the left maxillary alveolar ridge. The lesion measured nearly 10 cm in diameter and extended across the midline. The mass was friable, with a central area of ulceration and necrosis.

**Figure 1:**
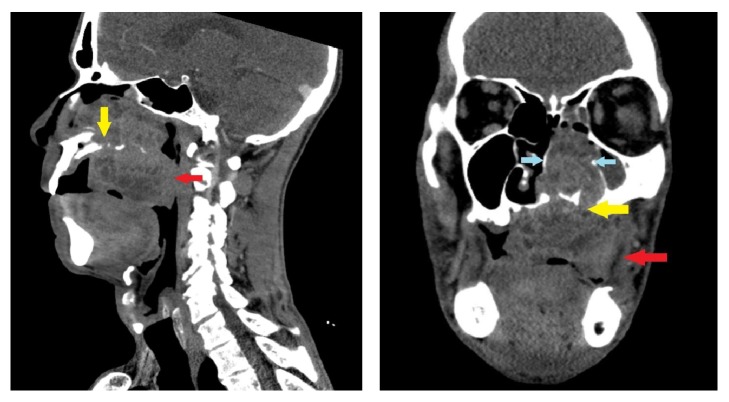
Sagittal and coronal contrast-enhanced CT images of the neck show a large, heterogeneously enhancing and locally aggressive mass centered at the hard palate to the left of midline. Mass erodes the hard palate (yellow arrow) and extends superiorly into the nasal cavity with extensive bony remodeling (blue arrows). Large exophytic component projects into the oral cavity (red arrow).

Panoramic radiograph showed erosion of the maxillary bone in the area of the lesion, and computed tomography (CT) demonstrated a 7.0 cm × 6.6 cm × 7.5 cm irregularly enhancing mass lesion centered on the left maxilla (Figure 1).

A punch biopsy was performed at an outside institution at the anterior superior portion of the lesion, and pathologic examination revealed oral mucosa consistent with a malignant glandular epithelium neoplasm. The lesion was poorly demarcated and consisted of glandular cells, primarily a monomorphous population of optically clear cells with central hyperchromatic and pleomorphic nuclei surrounded by clear cytoplasm. The neoplastic clear cells were arranged in nests, cords, and anastomosing trabeculae embedded in hyalinized, acellular, predominantly basophilic stroma. The overlying squamous mucosa was intact keratinized, acanthotic squamous epithelium. The neoplastic cells were periodic acid-Schiff (PAS) positive, which was abolished by diastase and mucicarmine negative. These results suggested that the clear cytoplasm was due to glycogen accumulation, not mucin production. Neoplastic cells were positive for pancytokeratin AE1/AE3, cytokeratin 7, smooth muscle actin, S-100, and p-40, which is consistent with myoepithelial differentiation. The tumor demonstrated a nodular infiltrative growth pattern, lacked overt ductal differentiation, and showed several areas of necrosis, including nests with comedo type necrosis. Although the cells were relatively bland cytologically, the infiltrative growth pattern suggested the diagnosis of myoepithelial carcinoma over myoepithelioma.

**Figure 2:**
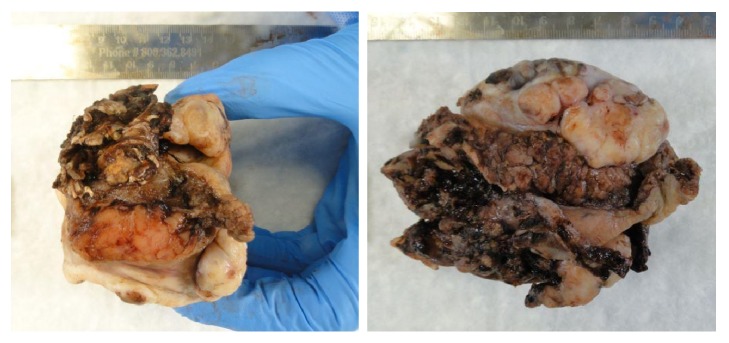
Representative images of near-total maxillectomy specimen measuring 9.2 × 6.5 × 6.5 cm overall. Examination revealed a 7.0 × 6.5 × 5.2 cm light tan, solid tumor replacing the majority of the hard palate.

At our institution, a near-total maxillectomy was performed which included the hard palate, soft palate, alveolar process, upper left maxilla, and nasal septum. Bilateral modified radical neck dissection, free tissue reconstruction, and tracheostomy were performed concurrently. The specimen size was 9.2 × 6.5 × 6.5 cm, and an exophytic tumor grossly involving the left hard palate invaded into the sinus cavities. Sectioning revealed a light tan, solid tumor, measuring 7.0 × 6.5 × 5.2 cm (Figure 2). It appeared to replace the majority of the hard palate and was adjacent to the lateral maxillary bone.

**Figure 3:**
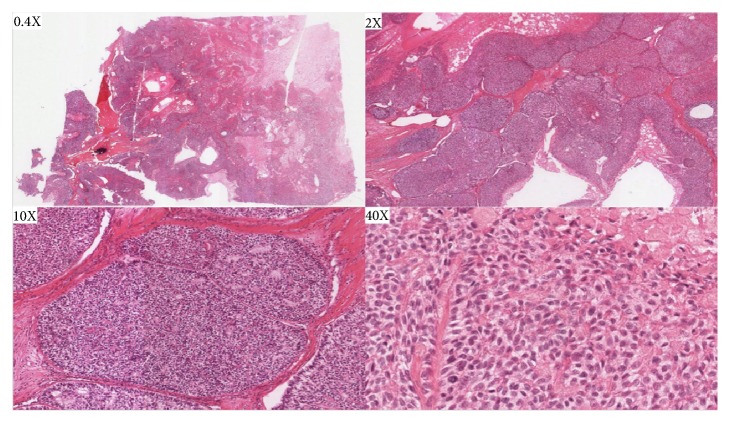
Representative images of oral mucosa with changes consistent with a malignant glandular epithelium neoplasm. The neoplastic clear cells were arranged in nests, cords, and anastomosing trabeculae embedded in hyalinized, acellular, predominantly basophilic stroma. The tumor demonstrated a nodular infiltrative growth pattern, lacked overt ductal differentiation, and showed several areas of necrosis, including nests with comedo type necrosis.

**Figure 4:**
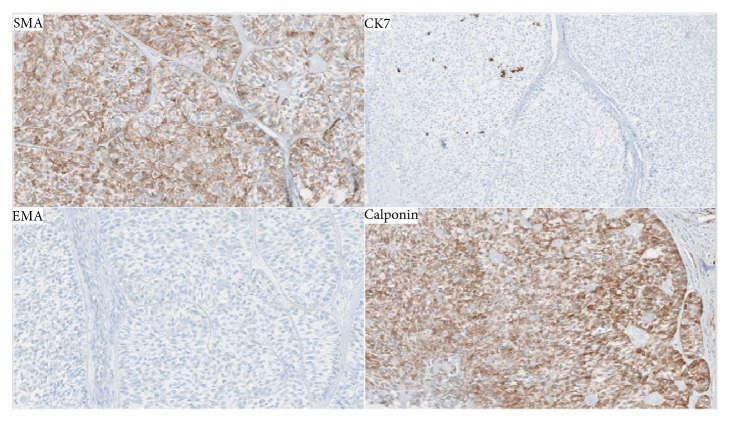
Immunohistochemical staining showing tumor positivity for SMA (smooth muscle actin) and calponin. CK7 was overall negative with a small fraction of the tumor showing patchy positivity. EMA was negative.

Histologic examination revealed similar findings to those described by the outside hospital: a poorly demarcated lesion consisting of nests of pleomorphic and hyperchromatic clear cells (Figure 3). Subsequent staining for p40, smooth muscle actin, CK7, EMA, and calponin confirmed a diagnosis of myoepithelial carcinoma, clear cell variant, moderately differentiated (Figure 4). The surgical margins were focally positive for invasive carcinoma. No lymphovascular or perineural invasion was noted. Tumor invasion of nearby structures included the cortical mandibular and maxillary bone, deep extrinsic muscle of the tongue (genioglossus, hyoglossus, palatoglossus, and styloglossus), maxillary sinus, and skin of the face. The lymph node dissection included 74 lymph nodes examined, and no metastatic disease was identified. These findings corresponded to a pT4aN0 pathologic staging.

**Figure 5:**
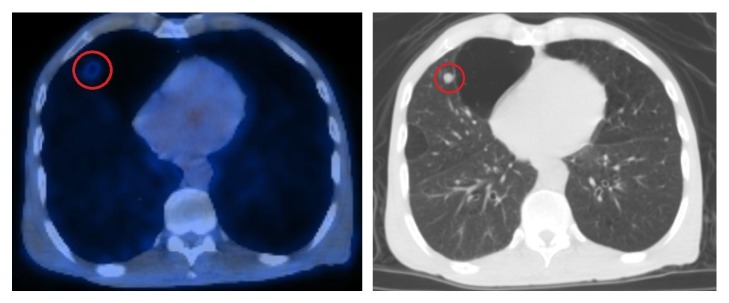
PET/CT shows a hypermetabolic spiculated lung nodule in the right middle lobe. There is background severe bullous emphysema.

Due to the advanced tumor stage (T4), the patient underwent postop radiation to the primary site and neck. Twelve weeks after radiation (seven months after the surgery), positron emission tomography/computed tomography (PET/CT) revealed mildly hypermetabolic bilateral pulmonary nodules that were concerning for metastatic disease (Figure 5). The patient was lost to follow up after this visit.

**Figure 6:**
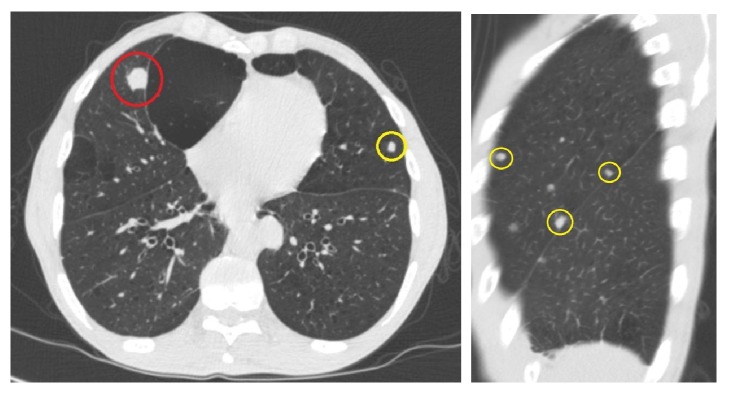
Noncontrast axial and sagittal CT images of the chest show interval enlargement of prior right middle lobe nodule (red circle) and multiple new nodules throughout the lungs (yellow circles).

**Figure 7:**
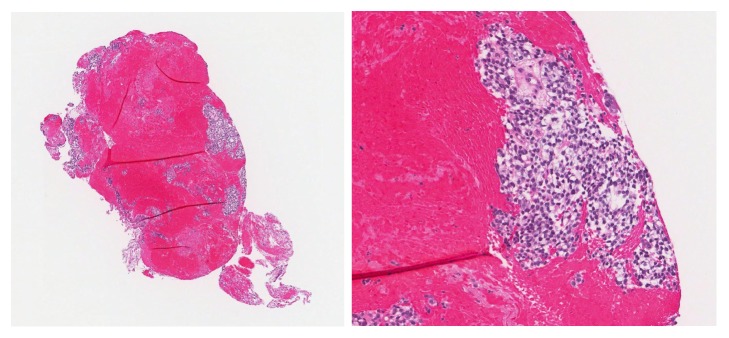
Representative low and high power images of bronchoscopic biopsies seventeen months later. The two lung nodules from the left lower lobe showed morphology consistent with metastatic CCMC.

At 17 months after resection of the neoplasm, the patient returned for follow-up. The patient denied any difficulty breathing, hemoptysis, or weight loss but was wearing a cervical collar due to report of a spine metastasis. Outside CT of the thorax showed bilateral pulmonary nodules of various sizes throughout all lung lobes (Figure 6). In comparison to the CT that first showed lung nodules, the nodules have increased in size and number. The largest nodule in the right lung was located in the right middle lobe, measuring 1.7 × 2.0 cm, while the largest nodule in the left lung was located in the left lower lobe, measuring 2.6 × 4.2 cm. No pathologically enlarged lymph nodes were identified within the mediastinum. Outside repeat PET/CT and MRI of the spine also confirmed likely metastasis to the C2 body. Bronchoscopic biopsies of two lung nodules from the left lower lobe showed morphology consistent with metastatic CCMC (Figure 7).

## 3. Discussion

Myoepithelial carcinoma is an uncommon neoplasm that arises from the salivary glands with the majority originating from the parotid gland and palate. It can be distinguished from its benign counterpart, myoepithelioma by its invasive and locally destructive behavior. Here, we present a rare case of this malignancy with metastasis to the lungs. Variants of tumor cell types include spindled, stellate, epithelioid, plasmacytoid, and clear cell [10, 11]. The diagnosis of myoepithelial carcinoma can be difficult often requires the assistance of an immunohistochemical panel [10, 12–14].

Immunohistochemical confirmation of myoepithelial differentiation involves positive stains for both cytokeratins and more than one myoepithelial marker as the differential of clear cell tumors is broad. In our case, the initial outside biopsy stained positively to pancytokeratin, S-100, and smooth muscle actin, which is consistent with myoepithelial differentiation. At our institution, staining with smooth muscle actin, p40, and calponin were positive. EMA and CK7 were negative. No intracellular mucin was detected on the histochemical stain mucicarmine. Cytoplasmic PAS staining was abolished with diastase treatment, indicating that the clear cytoplasm was at least in part due to glycogen accumulation rather than mucin production. Again, this is consistent with myoepithelial differentiation.

Most myoepithelial carcinomas arise from the major salivary glands, followed by minor salivary glands. Only a handful of cases have been reported to arise from the palate. Like other reported cases of myoepithelial carcinoma from the palate, this case presented as a painless mass [10]. The majority of cases are treated with radical surgical excision [10, 15], and the effects of chemotherapy and radiation have not been well studied. However, these treatment modalities are routinely used to treat systemic metastasis [15].

A review from 2002 found that 50% of clear cell myoepithelial carcinoma recurred following treatment, and 40% metastasized to the lung and scalp (n=10) [16]. Another study from 2015 examined 21 cases of CCMC and found that 52% of cases had recurrent or metastatic disease, with 5 cases to the lymph nodes, 7 cases invading the orbit, and 1 case each metastasizing to the neck soft tissues, liver, lungs, mediastinum, and thoracic vertebrae. This study also showed a mortality rate of 38% [17]. The prognosis for clear cell myoepithelial carcinoma is not well defined due to the low number of cases reported, but these studies suggest that CCMC is an aggressive neoplasm. Currently, several molecular point mutations such as HRAS, CTNNB1, and PIK3CA have been described and a 5 year survival rate of 94% with a variable rate of recurrence [18]. Thus, accurate diagnosis of CCMC and differentiation from other tumors of the intraoral cavity are important to determine prognosis and treatment.
